# Liver Hemorrhage Following Mechanical CPR With the Lund University Cardiopulmonary Assist System (LUCAS) Device: A Focused Case Report

**DOI:** 10.7759/cureus.61107

**Published:** 2024-05-26

**Authors:** Hussein Harb, Taimoor Munawar, Hasan Al-Obaidi, Zain Shehzad, Alice Sonnino

**Affiliations:** 1 Elective Rotations, Ross University School of Medicine, Bridgetown, BRB; 2 Internal Medicine, Jamaica Hospital Medical Center, New York City, USA; 3 Internal Medicine, Cleveland Clinic Florida, Weston, USA

**Keywords:** post-flight cardiac arrest, massive pulmonary embolism, lucas device efficacy, mechanical cpr, emergency resuscitation, liver damage, cardiac arrest management

## Abstract

Cardiac arrest is a leading cause of mortality globally, and mechanical CPR devices like the LUCAS system are designed to improve outcomes by enhancing consistency and reducing rescuer fatigue. However, this case report of a 76-year-old female who suffered cardiac arrest post-flight reveals significant complications associated with mechanical CPR. Despite achieving initial resuscitation, she developed extensive liver damage and additional complications, which ultimately led to her death. This case underscores the importance of precise training and strict adherence to guidelines when using mechanical CPR devices. It highlights that while these devices offer potential benefits, they also pose risks, especially for vulnerable patients, necessitating careful consideration and ongoing evaluation to optimize safety and effectiveness.

## Introduction

Cardiac arrest is a leading cause of death globally and prompts over a million CPR attempts annually in the United States alone [1]. Mechanical CPR devices such as the LUCAS aim to enhance the consistency and efficacy of CPR, ensure continuous compressions, and reduce physical strain on healthcare providers [2]. However, these devices raise concerns related to their effectiveness in real-world scenarios compared to manual CPR, as well as their potential to cause complications like visceral organ damage [3]. This case report presents the medical journey of a 76-year-old female who experienced a cardiac arrest after a long flight and highlights the potential complications linked with the use of mechanical CPR in emergency medical settings.

## Case presentation

A 76-year-old female with a history of hyperlipidemia, hypertension, and insulin-dependent diabetes was transported to the emergency department following a cardiac arrest after an 11-hour flight. EMS reported that she called for help due to shortness of breath, then suddenly collapsed, developing hypotension and pulseless electrical activity. In the field, Advanced Cardiac Life Support (ACLS) was initiated, including the application of an automated external defibrillator (no shock delivered), 30 minutes of CPR with a LUCAS device, and the administration of seven doses of epinephrine and five doses of midazolam, resulting in ROSC.

Upon ED admission, the patient was hemodynamically unstable, displaying hypotension (blood pressure of 58/32 mmHg), tachycardia (135 beats per minute), and tachypnea (33 breaths per minute), necessitating intubation and ventilation on 100% FiO2. Neurologically, she responded to pain, exhibited a positive corneal reflex, and had 3/5 bilateral upper extremity strength.

Lab findings included hyperglycemia (497 mg/dL), elevated BUN/Cr (25/1.6 mg/dL), hyponatremia (133 mmol/L), hypokalemia (2.7 mmol/L), an elevated anion gap (22.0), metabolic acidosis (bicarbonate 14 mmol/L), significant transaminitis (ALT/AST 908/809 U/L), lactic acidosis (18.96 mmol/L), and lymphocytic leukocytosis (13.3 x10^3/μL).

**Figure 1:**
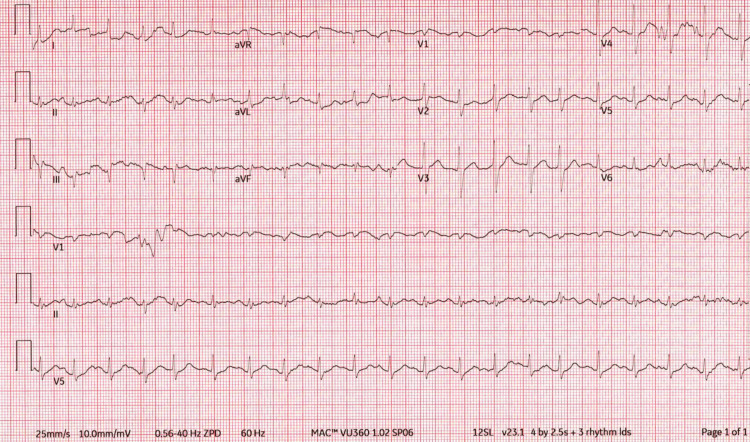
12-lead electrocardiogram showing sinus tachycardia with ST depressions in V5 and V6, occasional premature ventricular complexes, and non-specific ST and T wave abnormalities.

The electrocardiogram indicated sinus tachycardia with ST depressions in leads V5 and V6 (Figure 1). A chest X-ray showed right upper lobe consolidation. Head and abdominal computed tomography (CT) scans showed no acute changes; leg duplex ultrasonography excluded DVT. A CT chest identified extensive bilateral pulmonary emboli, and an echocardiogram revealed significant right heart dysfunction. Cardiology recommended mechanical thrombectomy for the pulmonary emboli.

The patient's elevated liver enzymes and creatinine were managed as shock-related liver injury and acute kidney injury, with interventions including an orogastric (OGT) tube, right upper quadrant ultrasound (ordered but not completed), lab monitoring, and hydration. Aspiration pneumonia was suspected based on leukocytosis and chest X-ray findings and treated with piperacillin-tazobactam and vancomycin.

The working diagnosis included cardiac arrest due to pulmonary embolism-related obstructive shock versus sepsis from bilateral aspiration pneumonia with non-ST elevation myocardial infarction (NSTEMI). Treatment included norepinephrine for hypotension, enoxaparin for pulmonary embolism, and pulmonary thrombectomy.

Post-thrombectomy, the patient developed leg swelling, toe cyanosis, and a pulseless right lower extremity. CT angiography suggested a thrombus in the inferior vena cava, but venography confirmed patency. She exhibited a significant hemoglobin drop, indicative of hemorrhage; a source was not identified in the lungs or gastrointestinal tract via bronchoscopy and negative stool guaiac, but guaiac-positive coffee-ground emesis from her OGT was noted.

**Figure 2:**
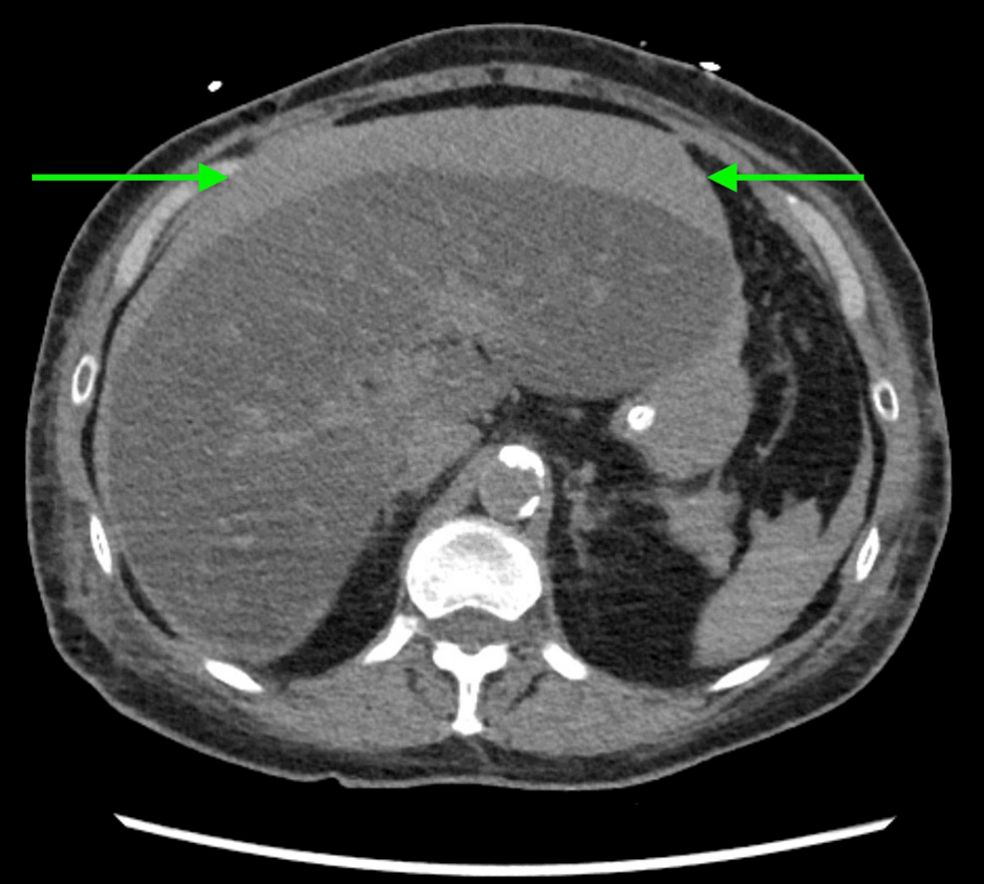
CT angiogram depicting perihepatic and capsular hemorrhage with hemoperitoneum layering in the bilateral paracolic gutters and pelvis. The perihepatic/capsular hemorrhage, as indicated by the green arrow, nearly encircles the liver, with areas up to 3 cm thick anteriorly.

Her condition worsened with rapid blood pressure drops and free fluid around the liver and left kidney observed on ultrasound. A CT angiogram identified a subcapsular liver hemorrhage (Figure 2), likely venous and possibly secondary to the previous day's CPR by the LUCAS device. Despite transfusions and continued pressurization, she developed a tense, distended abdomen and oliguria, prompting surgical liver packing.

**Figure 3:**
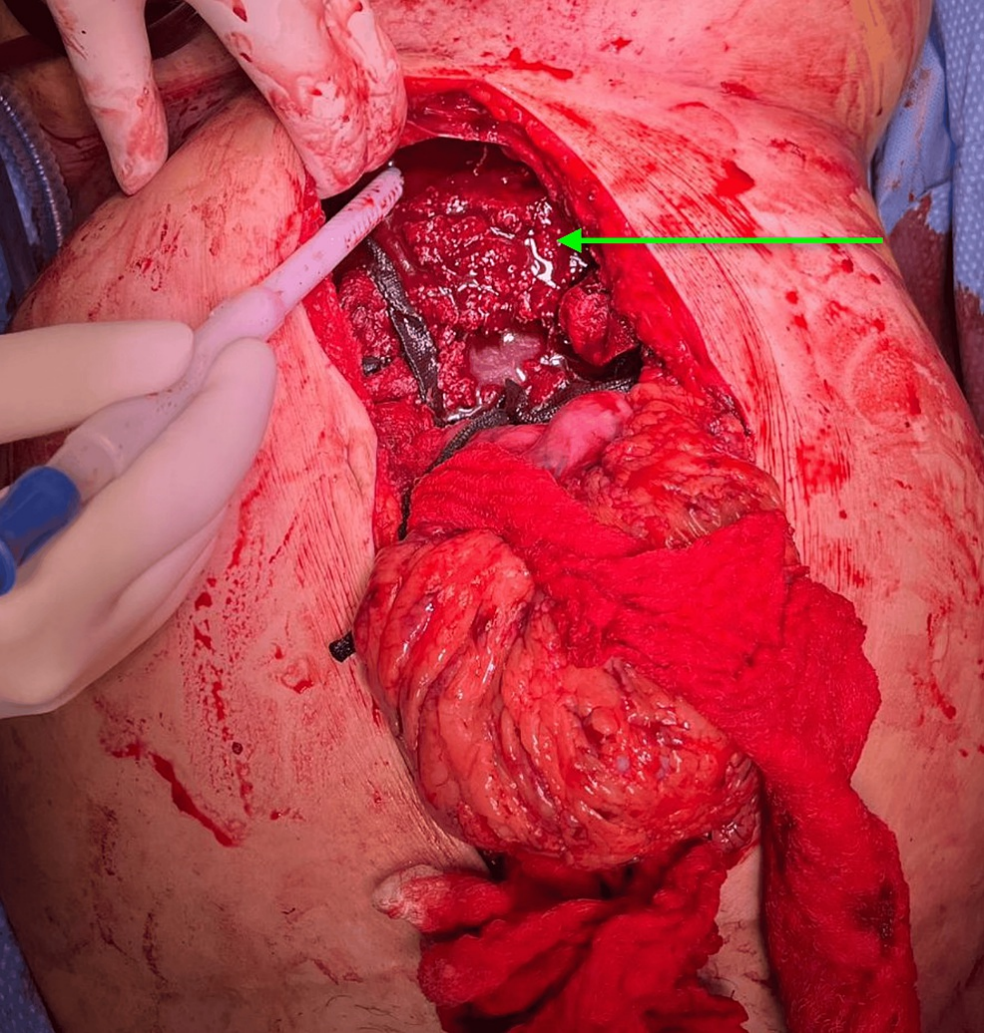
Intraoperative surgical procedure image showing extensive hemorrhage from the liver (green arrow) during the liver packing procedure.

Intraoperatively, immediately upon entering the abdomen, the surgeon was able to withdraw four liters of blood. The liver was found to be decapsulated across its entire anterior and superior surfaces. The liver capsule was densely adherent to the opposing peritoneum, resulting in massive hemorrhage (Figure 3). The likely cause of this liver injury was the use of a LUCAS CPR device the previous day. The liver was packed and planned for further resuscitation and surgery within 48 hours. However, the patient experienced a subsequent cardiac arrest in the OR and was pronounced dead after unsuccessful resuscitation efforts.

## Discussion

In the United States, approximately 350,000 cardiopulmonary resuscitation (CPR) attempts occur outside of hospitals annually, with an additional 750,000 attempts in hospitals totaling over a million CPR attempts each year [1]. Despite extensive educational efforts, the quality of in-hospital CPR often remains suboptimal, influenced by factors such as rescuer fatigue during prolonged resuscitation, interruptions in CPR for defibrillation or intubation, and inadequate compression depth and rate [4]. Mechanical chest compression devices, such as the LUCAS, offer a solution by delivering consistent, fixed-rate compressions by American Heart Association (AHA) guidelines [2]. These devices alleviate physical strain on rescuers, potentially improving compression fidelity and minimizing crowding in patient rooms, reducing exposure to aerosolized pathogens. Furthermore, they allow healthcare providers to perform other critical tasks during resuscitation efforts.

Despite evidence indicating that the LUCAS device delivers high-quality compressions consistently, where up to a third of manual compressions may be ineffective [5], studies have not demonstrated a significant difference in patient outcomes between manual and mechanical resuscitation [6-10]. Additionally, a meta-analysis has indicated that mechanical CPR may be associated with an increased risk of complications, such as rib fractures and cardiac or liver injuries, which could result from incorrect placement or migration of the device during use [11]. In this reported case, the patient likely suffered extensive liver damage leading to hemorrhage and eventual death due to improper application of the LUCAS device’s compressive mechanism. This underscores the necessity for ongoing, high-quality training of cardiac arrest teams on the correct usage of mechanical CPR devices. 

Furthermore, unlike manual CPR, setting up a mechanical CPR device requires additional time, potentially delaying the initiation of life-saving measures during the critical early phase of a cardiac arrest. Yet, studies suggest that with proper training, the time to initiate mechanical resuscitation can be reduced [6].

Current AHA guidelines recommend the use of mechanical CPR devices by trained personnel in situations where high-quality manual compressions are not feasible or could pose a risk to healthcare workers [12]. These guidelines may evolve as increased, high-quality training on mechanical CPR devices potentially results in improved resuscitation outcomes compared to manual ones. A notable case in 2013 involved a 68-year-old male who made a complete recovery from an out-of-hospital cardiac arrest after receiving 59 minutes of mechanical compressions via a LUCAS device, subsequently showing a favorable neurological outcome [13].

## Conclusions

This case report highlights the complexities of using mechanical CPR devices, like the LUCAS, in emergency medical scenarios. While such devices offer consistent compression rates and reduce physical strain on healthcare providers, they also present significant risks, as illustrated by the severe liver damage and subsequent death of a 76-year-old patient. This incident underlines the critical importance of proper training and strict adherence to guidelines when using mechanical CPR. It also emphasizes the need for ongoing research to optimize these devices for safety and effectiveness. As these technologies evolve and become more common in resuscitation efforts, continuous updates to clinical guidelines and thorough training for healthcare providers will be essential to balance the benefits of mechanical CPR with its potential risks, aiming to improve survival rates and neurological outcomes for cardiac arrest patients. 
